# Rearrangement of 1D Conducting Nanomaterials towards Highly Electrically Conducting Nanocomposite Fibres for Electronic Textiles

**DOI:** 10.1038/srep09300

**Published:** 2015-03-20

**Authors:** Joong Tark Han, Sua Choi, Jeong In Jang, Seung Kwon Seol, Jong Seok Woo, Hee Jin Jeong, Seung Yol Jeong, Kang-Jun Baeg, Geon-Woong Lee

**Affiliations:** 1 Nano Hybrid Technology Research Center, Korea Electrotechnology Research Institute (KERI) Changwon 642-120, Republic of Korea; 2 Department of Electrical Functionality Material Engineering, Korea University of Science and Technology (UST), Changwon. 642-120, Republic of Korea

## Abstract

Nanocarbon-based conducting fibres have been produced using solution- or dry-spinning techniques. Highly conductive polymer-composite fibres containing large amounts of conducting nanomaterials have not been produced without dispersants, however, because of the severe aggregation of conducting materials in high-concentration colloidal solutions. Here we show that highly conductive (electrical conductivity ~1.5 × 10^5^ S m^−1^) polymer-composite fibres containing carbon nanotubes and silver nanowires can be fabricated via a conventional solution-spinning process without any other treatment. Spinning dopes were fabricated by a simple mixing of a polyvinyl alcohol solution in dimethylsulfoxide with a paste of long multi-walled carbon nanotubes dispersed in organic solvents, assisted by quadruple hydrogen-bonding networks and an aqueous silver nanowire dispersion. The high electrical conductivity of the fibre was achieved by rearrangement of silver nanowires towards the fibre skin during coagulation because of the selective favourable interaction between the silver nanowires and coagulation solvents. The prepared conducting fibres provide applications in electronic textiles such as a textile interconnector of light emitting diodes, flexible textile heaters, and touch gloves for capacitive touch sensors.

Highly conducting fibres containing carbon nanotubes (CNTs) or graphene nanosheets are of great interest for various applications that range from composites for vehicles to flexible electronic (e)-textiles^1^,^2^,^3^,^4^,^5^,^6^,^7^. In the solid state, highly conductive fibres can be spun from vertically aligned CNTs^8^,^9^,^10^,^11^,^12^ or from an aerogel of CNTs formed in a chemical-vapor-deposition reaction chamber^13^,^14^. The solution-spinning method, which is used to create most synthetic fibres, has been used to form CNT-based fibres from CNT dispersions stabilised with surfactant solutions^15^,^16^ and super acids^17^,^18^. However, the development of liquid-state spinning of highly conducting polymer composite still faces challenges owing to difficulties in dispersing large amounts of conducting materials with high aspect ratios in a viscous polymer solution. Although the conducting composite fibres can be fabricated by using highly functionalised CNTs, severe functionalisation of nanotubes is disadvantageous because the overall properties of the resulting fibres can suffer because of the decreased electrical conductivity of the CNTs^19^,^20^. Recently, we have reported that with the assistance of quadruple hydrogen bonding (QHB) networks, highly conductive CNTs and graphene nanosheets can be dispersed in an organic solvent without using a dispersant^21^. However, diversified applications of nanocarbon-based conducting pastes in the conducting fibre can be limited by the incompatibility with many kinds of polymer matrix materials dissolving in different solvents. To solve this problem, we need to design the chemical structure of QHB motifs introducing in nanocarbon materials. Moreover, polymer-composite fibres with only nanocarbon material do not meet the requirement for conducting fibres for applications in electronics because of the high junction resistance between conducting materials. Until now, highly loaded polymer-composite fibres have been fabricated by injecting a surfactant-based CNT dispersion into a polymer solution to form a coagulation solution^15^. Even when highly conductive and expensive single-walled CNTs are used, these processes have limited ability to obtain conductive fibres with high electrical conductivity (over 10000 S m^−1^) because it is difficult to minimise the junction resistance between nanotubes. In this context, highly conductive one-dimensional (1D) metal nanowires such as silver nanowires (AgNWs) can be a good conducting additive in terms of percolation with 1D CNTs, provided that we can prepare a mixed solution of a polymer and conducting particles that contain a large amount of conducting material.

Here we show a straightforward method to fabricate highly conductive polymer-composite fibres via direct solution spinning with well-dispersed CNT/AgNW/polymer pastes for electronic textiles. Long multi-walled CNTs (LMWNTs) were modified with QHB motifs (QHB-LMWNTs) containing polypropylene glycol moieties for high dispersion even in the presence of water. The wet-spinning dope was prepared by direct mixing of a QHB-LMWNT dispersion in an organic solvent, an aqueous AgNW dispersion, and polyvinyl alcohol (PVA) in dimethylsulfoxide. During coagulation in the solution, rearrangement of the AgNWs towards the surface layer of the fibre, combined with interconnection with LMWNTs, led to an electrical conductivity of the fibre that was as high as 1.5 × 10^5^ S m^−1^. The local population of AgNWs in the fibre was confirmed by removing PVA via so-called chemical scribing of the fibre surface.

## Results

### Fabrication of conducting pastes for fibre spinning

**Figure 1:**
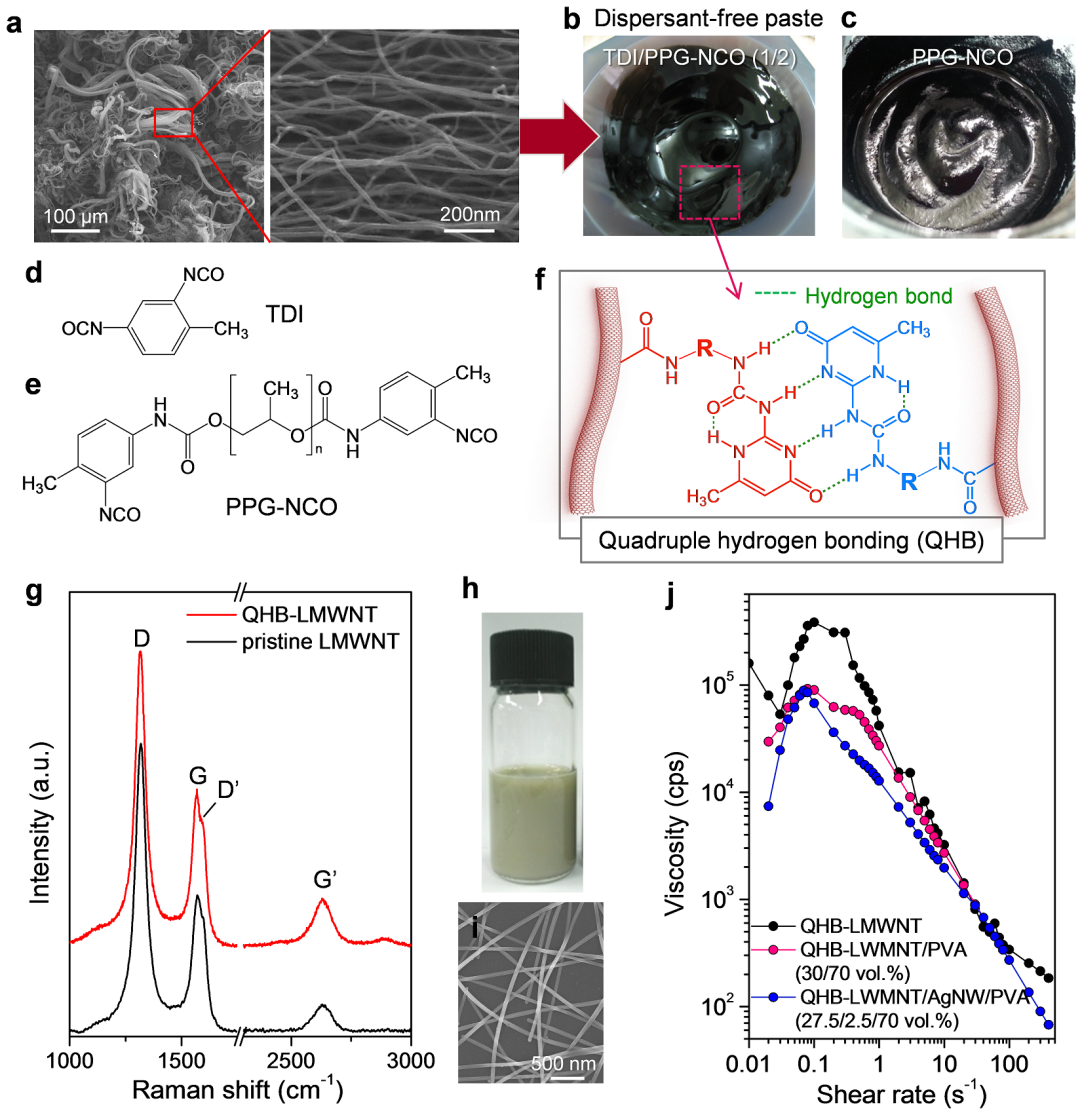
Preparation of spinning dopes. (a) Field-emission scanning electron microscope (FESEM) image of LMWNTs. (b), (c) Photo images of dispersant-free LMWNT pastes assisted by quadruple hydrogen bonds formed via coupling reaction with (b) TDI–PPG-NCO and (c) PPG-NCO. (d), (e) Chemical structures of TDI and PPG-NCO, respectively. (f) Chemical structure of quadruple hydrogen-bonding motif. R indicates the chemical structure of diisocyanate compounds. (g) Raman spectra of pristine LMWNTs and QHB-LMWNTs. (h) Photo image of AgNW dispersion. (i) FESEM image of AgNWs. (j) Plots of viscosity vs. shear rate for the QHB-LMWNT paste, QHB-LMWNT/PVA mixture, and QHB-LMWNT/PVA/AgNW mixture.

In this study, commercially available and cheap LMWNTs (over 100 μm in size) were used as a conducting-network material, which is useful to enhance the contact between conducting materials in the PVA matrix (Figure 1a). Moreover, their high aspect ratio and the hydrophobicity of the nanotube surface provided an advantage to modulation of the arrangement of conducting materials during the coagulation process. However, it is usually difficult to disperse LMWNTs in organic solvents without high functionalization and shortening of nanotubes by mechanical chopping in the absence of dispersant molecules. Therefore, before mixing LMWNTs with PVA, the LMWNTs were functionalised with QHB motifs (QHB-LMWNTs) to fabricate a well-dispersed LMWNT paste, as previously reported^21^. QHB-LMWNTs were synthesized in three steps from LMWNTs functionalised with carboxylic acid groups (LMWNT-COOH), diisocyanate compounds, and 2-amnio-4-hydroxy-6-methyl- pyrimidine (AHMP) (Figures S1–S3). Here, a mixture of toluene diisocyanate (TDI) and toluene 2,4-diisocyanate-terminated polypropylene glycol (PPG-NCO) (1:2 weight ratio) were reacted with LMWNT-COOH to enhance the dispersion stability of QHB-LMWNTs in the PVA matrix (Figure 1b–f). On its own, PPG-NCO does not promise the stable dispersion of LMWNTs in an organic solvent because of an equal reactivity of isocyanate groups in PPG-NCO (Figure 1c), which can covalently bridge the nanotubes and minimise the QHB introduction. Thus, TDI molecules with asymmetrically reactive isocyanate groups are necessary to attach a sufficient amount of QHB motifs. More importantly, the defect formation of LMWNTs during QHB attachment was minimised, as demonstrated by the Raman spectra (Figure 1g)^22^. The ratio of the intensities of the D band and G band (*I*_D_/*I*_G_) did not increase after QHB attachment, which means that most of the QHB motifs were introduced at the ends of the CNTs because of the high reactivity of the CNT end cap (Figure 1g)^23^. The prepared QHB-LMWNTs were easily dispersible in *N*-methyl pyrrolidone (NMP) or dimethylformamide (DMF) in the form of a paste (10 wt% solid content), which was very miscible with the PVA solution in dimethylsulfoxide (DMSO) with simple stirring (QHB-LMWNT/PVA paste). A certain amount of an aqueous AgNW suspension (0.5 wt%) stabilised by polyvinylpyrrolidone (PVP) (0.07 wt%) was added to this mixture (Figure 1h, i) and it was stirred mechanically to form the uniform paste (QHB-LMWNT/PVA/AgNW paste). The ratio of AgNWs to LMWNTs in the paste was controlled to vary from 0 to 13 vol%, and we did not observe any aggregation of the nanoparticles. To confirm that the prepared paste formed a stable dispersion in the presence of PVA, LMWNT, and AgNWs, we investigated the dependence of the viscosity of the pastes on the shear rate (Figure 1j). In the QHB-LMWNT and QHB-LMWNT/PVA paste, we observed shear thickening in the low-shear regime even at concentrations below 10 vol%, which clearly indicates that the QHB-LMWNTs were interconnected through strong interactions among the LMWNTs functionalised with QHB motifs in DMSO. However, we did not observe shear thickening in the QHB-LMWNT/PVA/AgNW mixed paste, which indicates that the QHBs were dissociated by water molecules from the aqueous AgNW suspension. PPG moieties introduced into the QHB motifs then enabled preparation of the stable mixture paste in the presence of water. Moreover, the dissociated QHB motifs may have also interacted with the hydroxyl groups in PVA.

### Fibre spinning and electrical properties of fibres

**Figure 2:**
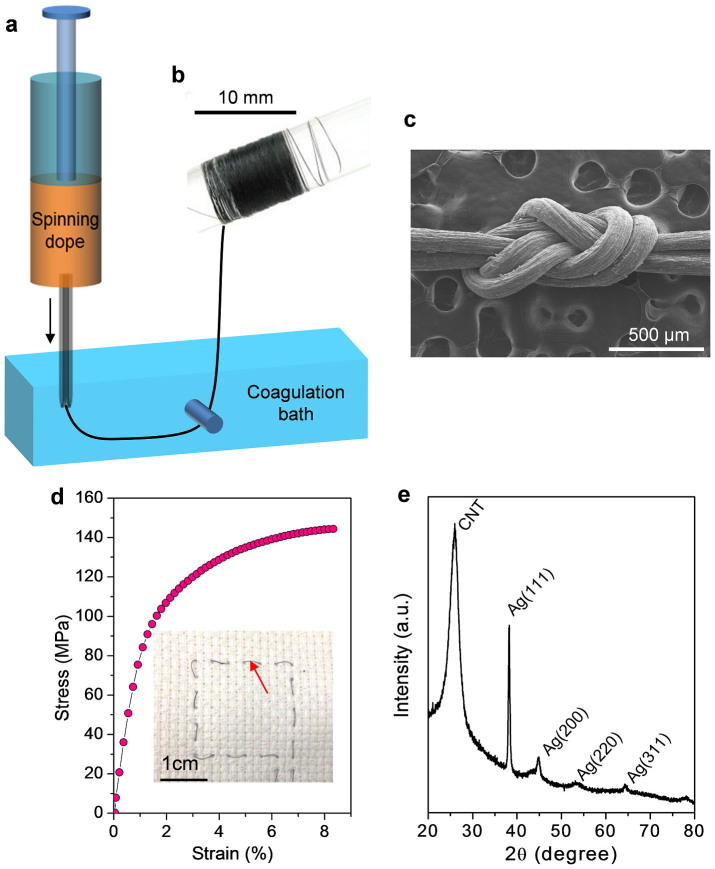
Fibre spinning and characterisation. (a) Schematic illustration of wet spinning process from a stable spinning dope containing LMWNTs, AgNWs, PVA, and solvents without dispersant molecules. (b) Photo image of fibre that was wet-spun from paste of QHB-LMWNT/PVA/AgNW mixture. (c) FESEM image of the fibre tied into a figure-eight splice knot. (d) Stress–strain plot of QHB-LMWNT/PVA/AgNW fibre containing 3.8 vol% of AgNWs. Inset photo image shows a pattern knitted in the fabric using a QHB-LMWNT/PVA/AgNW fibre indicated by red arrow. (e) X-ray diffraction pattern of the QHB-LMWNT/PVA/AgNW composite fibre.

We could easily fabricate the conducting fibres with QHB-LMWNT/PVA and QHB-LMWNT/PVA/AgNW mixed pastes via wet spinning, using methanol as a coagulation solvent (Figure 2a and b). The spun fibre could be tied into a figure-eight splice knot, as shown in Figure 2c, which means that it can be used in fabric applications. Furthermore, the tensile strengths of the fabricated composite fibres with a diameter of 55 μm ranged from 140 to 160 MPa, and the failure strains ranged from 8 to 10% (Figure 2d). The tensile strength of the fabricated composite fibre is much lower than that (over 1 GPa) of pure single-walled CNT (SWCNT) fibres prepared by using SWCNT liquid crystal paste^18^. However, it is much higher than that (below 10 MPa) of other polymer composite fibre containing over 30 wt% CNTs^20^. In a highly loaded system, the tensile strength decreases dramatically because of the less uniform distribution of nanotubes in the polymer matrix.

In all cases, the volume fraction of the nanotubes and AgNWs was maintained at ~30 vol% by adjusting the ratio of QHB-LMWNTs to AgNWs used in the spinning solution. By increasing the amount of QHB-LMWNTs, it was difficult to solidify the as-spun fibre in methanol because of the affinity between the QHB motifs and polar solvents. Dissociated QHB motifs in methanol could interact with methanol by hydrogen bonding. In cross-sectional images (Figure S4), we observed well-distributed LMWNTs or AgNWs. In particular, in the QHB-LMWNT/PVA/AgNW hybrid fibres, AgNWs were observed on the fibre surface through a charging effect under the electron beam of the field-emission scanning electron microscope (FESEM). The X-ray diffraction pattern of the hybrid fibre also shows the co-existence of LMWNTs and AgNWs in the fibre (Figure 2e).

**Figure 3:**
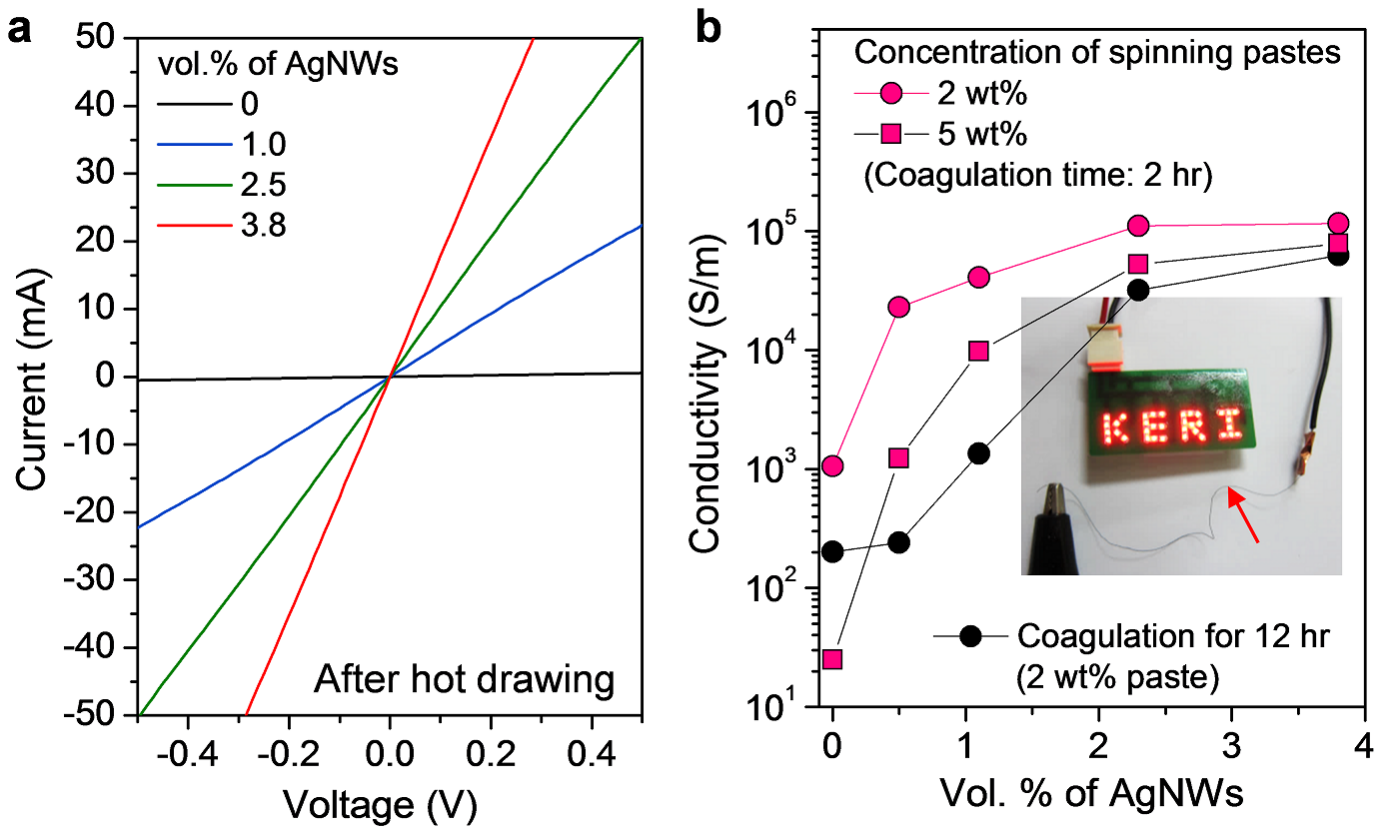
Electrical properties of conducting fibres. (a) I–V plots of composite fibres with increasing volume fraction of AgNWs. (b) Electrical conductivities of the spun fibres as functions of AgNW content for various paste concentrations and coagulation times. The inset photo image shows that the prepared conductive fibre (indicated by arrow) can be used as an interconnector to light 36 pieces of light-emitting diode lamps of a 15 V display.

Figure 3a shows the *I*–*V* characteristics of the fibres, and the current flow increased by adding more AgNWs. Figure 3b shows the electrical conductivities of the composite fibres as functions of the amount of AgNWs. The conductivity of the as-spun QHB-LMWNT/PVA composite fibre was 100 S m^−1^, which was enhanced to 1200 S m^−1^ through thermal drawing at 150°C. However, this value does not meet the requirement for a conducting fibre that serves as the interconnecting electrode or a fibre for e-textile applications. Most importantly, the conductivity of the fibre was dramatically increased from 1200 to 20000 S m^−1^ by adding 0.5 vol% of AgNWs; it reached 150,000 S m^−1^ in the composite fibre containing 3 vol% of AgNWs. However, The PVA composite fibre containing only 0.5 vol% AgNWs without QHB-LMWNTs was electrically insulating, and more AgNWs made the fibre electrically conducting. This means that 0.5 vol% AgNWs in the fibre are separated in PVA matrix. Therefore, our results indicate that small amounts of AgNWs in the composite fibre can be percolated in the presence of large amounts of QHB-LMWNTs. The conductivity even improved by 10% through thermal drawing at 150°C, which was more dominant at low loading of AgNWs. The electrical conductivity of the fibre was also dependent on the concentration of the spinning dope and the coagulation time. By increasing the paste concentration and the coagulation time, the maximum conductivity was reduced. A light-emitting diode lamp can be lit by preparing a conducting fibre as the flexible interconnector (Figure 3b, inset).

### Mechanism of high electrical conductivity

The dependency of the high electrical conductivity on the concentration and the coagulation time was investigated by characterising the fibre surface to explore its inner structure via a chemical scribing. In fact, AgNWs dispersed in a PVP solution were more favourable for the coagulation solvent, methanol, than hydrophobic LMWNT surfaces. This leads to the hypothesis that AgNWs can be located in the surface layer of a fibre, while LMWNTs will be flocculated at the core. To confirm the location and population of AgNWs in the fibre, the composite fibres were chemically scribed by first dipping in DMSO and a DMSO/methanol (1:1 v/v) mixture for 5 min, followed by immersion in methanol. By using DMSO, the thick surface layer of the fibre was removed (harsh scribing), while only PVA was removed by the DMSO/methanol mixture (mild scribing) in terms of the solubility parameter.

**Figure 4:**
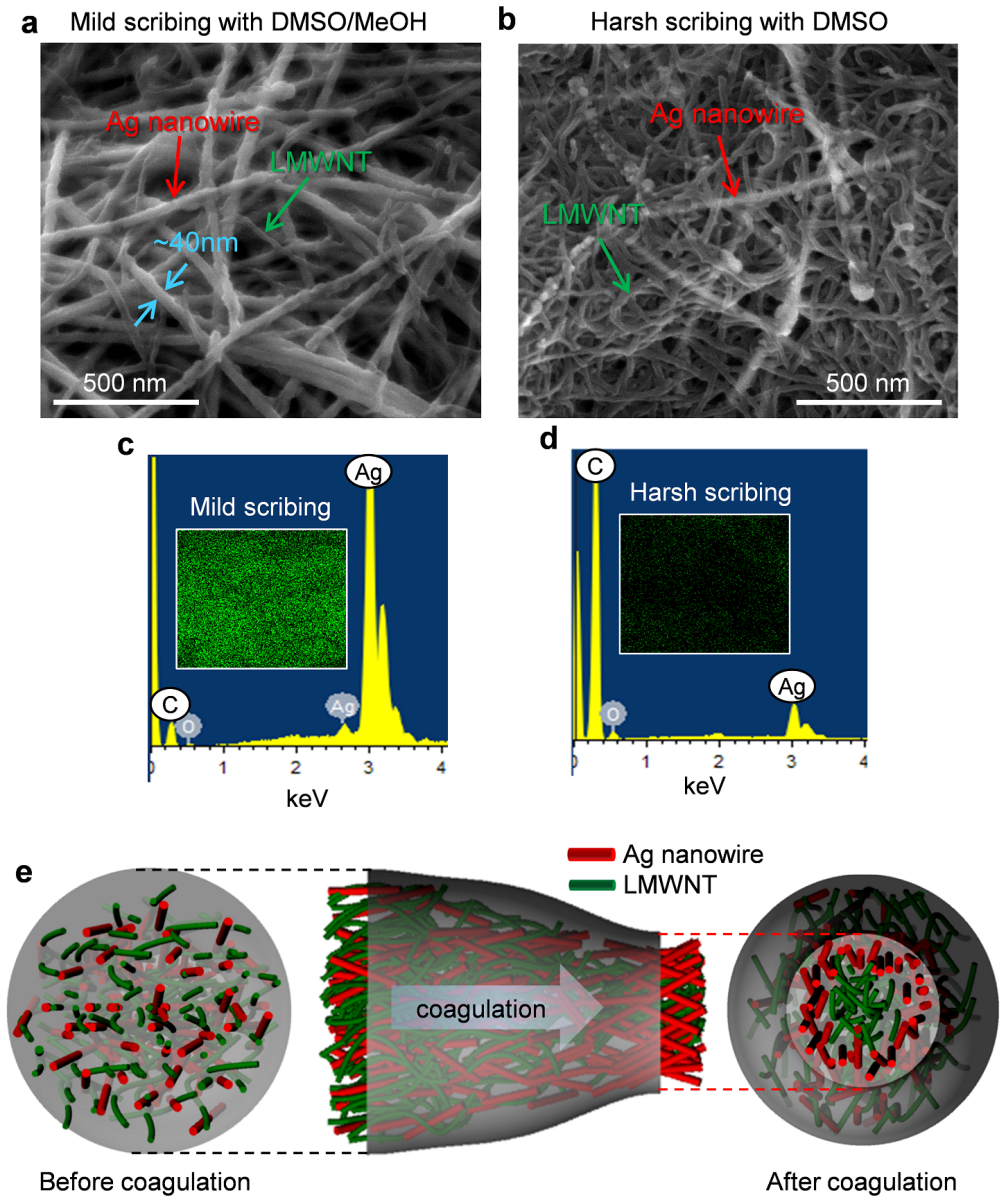
Characterisation of fibre surfaces. (a), (b) FESEM images of QHB-LMWNT/PVA/AgNW (27.5:70:2.5 v/v/v) fibres prepared with 2 wt% paste after (a) mild scribing with DMSO–methanol mixture and (b) harsh scribing with DMSO. c, d) EDX data of QHB-LMWNT/PVA/AgNW (27.5:70:2.5 v/v/v) fibres after c) mild scribing and (d) harsh scribing. Inset images in (c) and (d) show EDX mapping images of Ag atoms. (e) Schematic illustration of the distribution of AgNWs and LMWNTs in the fibre before and after coagulation.

Figure 4a and b show the FESEM images of the surfaces for the fibres (2.5 vol% AgNWs) prepared with the 2 wt% paste after chemical scribing. The surface image after mild scribing with the DMSO/methanol mixture clearly demonstrates that in the shallow fibre surface, AgNWs were the dominant constituents, while in the deeper surface layer, mostly LMWNTs were observed after harsh scribing. Energy-dispersive X-ray (EDX) spectra of the chemically scribed fibres also showed the population of AgNWs in the fibre surface (Figure 4c and d). Most importantly, the electrical conductivity of the fibre after harsh scribing dramatically decreased to ~10,000 S m^−1^. Therefore, most of the AgNWs that were homogeneously dispersed in an as-spun fibre before coagulation were moved to the surface layer during coagulation, as illustrated in Figure 4e. However, for the fibre that was fabricated with the 5 wt% paste, we could not observe the different populations of AgNWs in the shallow and deeper surfaces of the fibre after chemical scribing (Figure S5). As known, in agreement with the rules of phase transition kinetics, precipitation in a spinning bath occurs owing to loss of solubility of the spinning solution caused by partial decomposition^24^. In another words, addition of a precipitate causes desolvation of the polymer, an increase in the number of linkages, loss of mobility, and transition to the solid state. The first kinetic stage is the diffusion of precipitant and its interaction with the solvated materials. At low concentrations of the polymer and weak desolvating power, the polymer solvate does not completely decompose and the fibre precipitates in the form of a highly swollen gel. This indicates that some solvate can be diffused out from the polymer solution if the fillers are favourable for the coagulation solvent. In our system, AgNWs are favourable for the coagulation solvent, methanol, while PVA and CNTs can be easily desolvated by the coagulation solvent. Therefore, the local abundance of AgNWs in the surface was dependent on the solid content of LMWNTs in the paste because it limits the diffusion of AgNWs into the coagulation solvent in highly concentrated pastes during solidification. This result clearly corresponds to the low electrical conductivity of the fibres fabricated with high-concentration pastes (Figure 3a). Moreover, by increasing the coagulation time, more PVA was found in the fibre surface (Figure S6), which may also be the reason for the decrease in conductivity after longer coagulation. To further clarify the rearrangement of AgNWs during coagulation in the solution, the spinning dope containing 2 vol% of AgNWs was bar-coated to form a composite film on the glass substrate. The electrical conductivity of the film was smaller (58,000 S m^−1^) than that (~10^5^ S m^−1^) of the corresponding fibre. To confirm the population of the AgNWs in the film surface, the films were chemically scribed using the same procedure carried out for fibres. It should be noted that we could not observe the local abundance of AgNWs in the surface while they were percolated (Figure S7). This result also indicates that the AgNWs were reorganized during coagulation in the solution to form the solid fibre structure.

### E-textile applications

**Figure 5:**
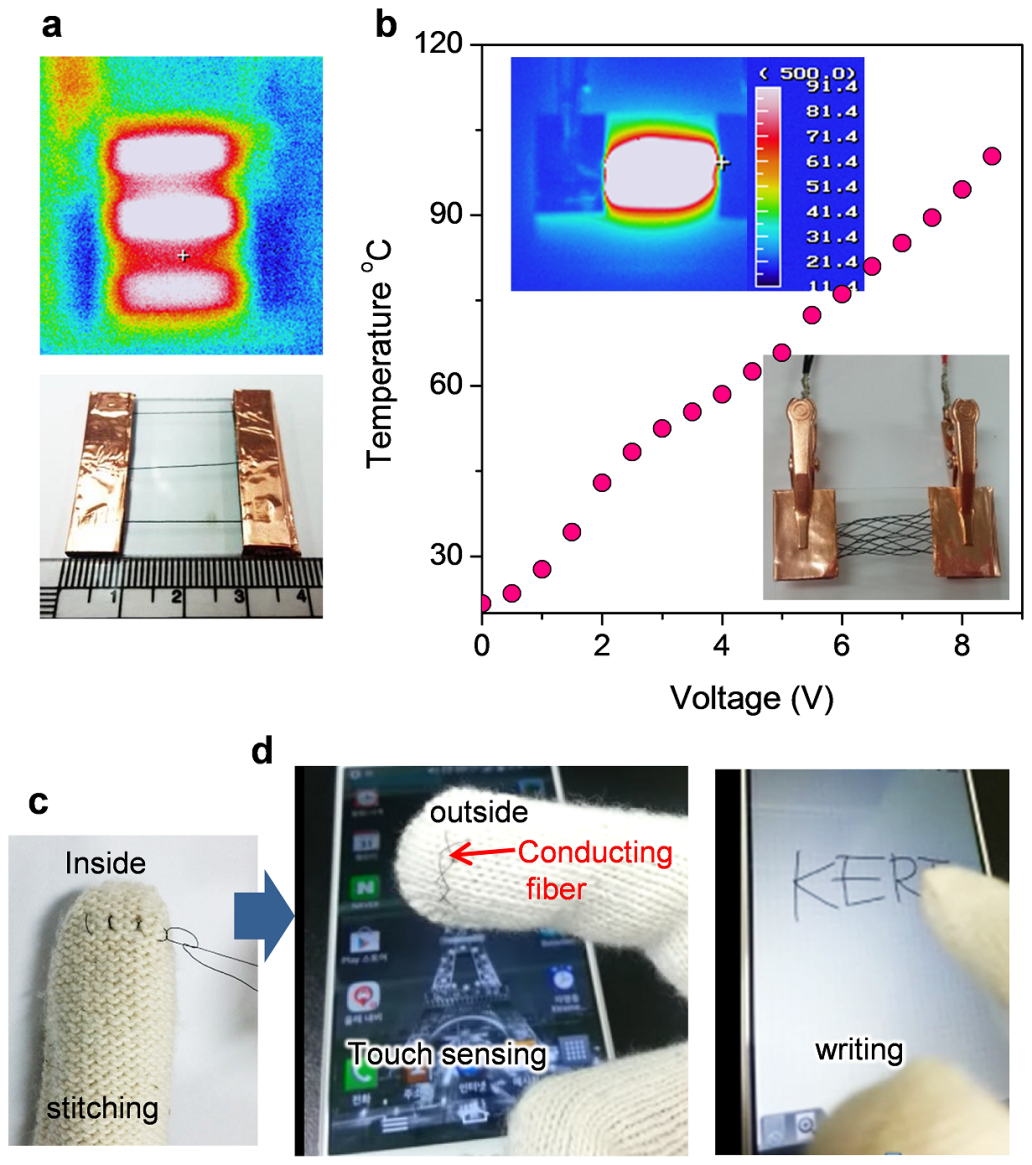
E-textile applications. (a) Infrared thermal image of individual conducting fibres (optical photo image) at an input voltage of 5 V. (b) Relationship between the temperature of the woven fabric (lower inset) and the input voltage. The upper inset image shows the temperature distribution recorded by the thermal infrared imaging camera. (c) The conducting composite fibre was stitched into the glove. (d) Photo image showing touch screen sensing and writing with the glove stitched with the conducting composite fibre.

The good properties of QHB-LMWNT/PVA/AgNW hybrid fibres make them capable of being woven conductive fabrics. Figure 5a shows the stable heating behavior of individual fibres even at a low input voltage of 5 V. The woven fabric was heated with a uniform temperature distribution for the most part (Figure 5b). Increasing the input voltage caused the temperature of the fabric to linearly increase to more than 100°C, which demonstrates the applicability of our composite fibres as textile heaters. In addition, to demonstrate the application of our conductive composite fibres in wearable devices, we stitched the conductive fibre into the index fingertip of the glove to work the capacitive-type touch screen panel used in smart phones and tablets (Figure 5c). Without conducting fibres, the capacitive touch panel does not work because the electrical current does get passed through the glove to the touchscreen (Movie S1). However, our conductive composite fibres stitched in the glove allowed electric current to flow through its fibres from touch screen to skin (Figure 5d). Moreover, polymer composite fibres can minimise the hazardous release of nanomaterials from the conducting fibre, which is one of the most important points to consider for use of conducting fibres in wearable devices contacting directly with human skin.

## Discussion

In summary, we demonstrated that highly conductive polymer-composite fibres can be fabricated by direct wet spinning of a LMWNT dispersion stabilised by QHB networks in a polymer solution containing AgNWs. Our strategy for producing highly conductive polymer-composite fibres was to reorganize highly conductive AgNWs in the fibre surface layer during coagulation as well as to well disperse LMWNTs in the polymer matrix that could connect the nanowires. Along with these experiments, we proved that the controlled location of highly conducting 1D materials in a polymer fibre matrix can assure the production of highly conductive fibres despite the volume fraction (70 vol.%) of the polymer matrix. We propose that our strategy can be applied to fabricate conducting fibres with CNTs, graphene nanosheets, metal nanowires, metal nanoparticles, etc. As application demonstrations, these composite fibres are used to fabricate flexible interconnector of LED lamps, textile heater, and textile conductors in touch gloves.

## Methods

### Synthesis of the long multi-walled carbon nanotubes (LMWNTs) with quadruple hydrogen bond (QHB) networks (QHB-LMWNTs)

To fabricate the spinning dope with QHB-LMWNTs, LMWNTs (purchased from Hanwha Chemical) functionalised with carboxylic acid groups (LMWNTs-COOH) were reacted with a mixture (1:2 w/w) of toluene diisocyanate (TDI, Aldrich) and toluene 2,4-diisocyanate terminated polypropylene glycol (PPG-NCO, Aldrich), and then with 2-amino-4-hydroxy-6-methyl-pyrimidine (AHMP, Aldrich), as previously reported (Figure S1). The LMWNTs were functionalised with carboxylic acid using a mixture of sulfuric acid and nitric acid (7:3 v/v) at a low temperature of 50°C for 12 h to minimise the defect formation in the starting materials. First, 3.0 g of LMWNTs-COOH were dispersed in 1 L DMF by bath sonication for 1 h under argon purging. The isocyanate groups were attached using the coupling reaction by adding an excess amount (8 mL) of the TDI–PPG-NCO mixture (1/2 w/w) to the LMWNT-COOH suspension, following by heating at 50°C for 24 h. The unreacted isocyanate compounds were removed by washing with DMF, what is used for the reaction, and vacuum filtration. Next, 3 g of the isocyanate-modified LMWNTs obtained was redispersed in 1 L of DMF by bath sonication. AHMP (3.6 g) and 8 mL of triethylacetate (catalyst) were added to the solution to attach the 2-ureido-4[1H]pyrimidinone moieties, and the solution was heated at 100°C for 20 h under magnetic stirring. The modified LMWNTs were the purified by centrifugation and vacuum filtration to remove the unreacted agents.

### Fabrication of conducting fibre by solution spinning and its applications

The conducting LMWNT pastes for solution spinning were prepared simply by mixing the QHB-LMWNT paste in NMP with the PVA solution in DMSO using a rotary mixer. A certain amount of aqueous AgNW suspension (0.5 wt%) stabilised by polyvinylpyrrolidone (0.07 wt%) (purchased from Nanopyxis) was added to the mixture. These QHB-LMWNT/PVA/AgNW pastes were injected at 10 mL min^−1^ through 22-guage (nominal inner diameter: 0.4 mm) needles into the rotating methanol by varying the coagulation time. The textile heaters were fabricated in a two-terminal side-contact configuration. The DC voltage was supplied by a power supply to the film heater through the fibre end. The temperature of the textile heater was measured using an IR thermal imager and a thermocouple.

### Characterisation

The surfaces and cross-sectional morphologies of the samples were imaged by field-emission scanning electron microscopy (FESEM; S4800, Hitachi). The structural characteristics of the LMWNTs and AgNWs in the fibre were investigated by confocal Raman spectrometry (NTEGRA Spectra, NT-MDT) with an excitation wavelength of 633 nm. The chemical states of the modified LMWNTs and AgNWs were determined by X-ray photoelectron spectroscopy (XPS) and Fourier-transform infrared spectroscopy (FT-IR) using a Multilab2000 (Thermo VG Scientific Inc.) spectrometer with monochromatised Al K as the X-ray excitation source and an FT-IR spectrometer (Model 4200UP, JASCO), respectively. The quantities of organic moieties in each synthesis step were confirmed by thermogravimetric analysis (TGA Q500, TA Instruments). The shear viscosities of the conducting pastes were measured using a Brookfield viscometer (DV-111 ultra). The electrical conductivity of the fibre was measured by the two-probe resistivity-measuring instrument with uniform 2 mm spacing at room temperature. Mechanical properties were measured using a thermomechanical analyzer at a rate of 500 μm min^−1^. The conducting fibre with a diameter of 55 μm was mounted on aperture cards (1 cm length window) and fixed with commercial superglue. The heating behaviour of the fibre was observed by using an IR thermal imager (TH9100, NEC San-ei Instrument, Ltd.) and a thermocouple (CENTER300, Center technology corp.).

## Author Contributions

J.T.H. conceived and supervised the experiment and wrote the manuscript. S.C. and J.I.J. carried out the experiment for preparation of spinning dopes and fibre spinning. S.K.S. and J.S.W. performed the characterisation of the fibre. H.J.J., S.Y.J. and K.-J.B. analyzed the data. G.-W.L. supervised the experiment and discussed the data. All authors discussed the results and commented on the manuscript.

## Supplementary Material

Supplementary Information Supplementary information

Supplementary Information Supplementary movie
