# Supplementary material for: Rearrangement of 1D Conducting Nanomaterials towards Highly Electrically Conducting Nanocomposite Fibres for Electronic Textiles
Source: Sci Rep. 2015 Mar 20;5:9300. doi: 10.1038/srep09300 (PMC4366813; doi:10.1038/srep09300)
Supplement: Supplementary Information [file srep09300-s1.doc]

**Supplementary Information**

**Rearrangement of 1D Conducting Nanomaterials towards Highly Electrically Conducting Nanocomposite Fibres for Electronic Textiles**

Joong Tark Han,^1, 2^ Sua Choi,^1^ Jeong In Jang,^1^ Seung Kwon Seol,^1, 2^  Jong Seok Woo,^1^ Hee Jin Jeong,^1^ Seung Yol Jeong,^1^ Kang-Jun Baeg,^1^ and Geon-Woong Lee^1^

^1^ Nano Hybrid Technology Research Center, Korea Electrotechnology Research Institute (KERI) Changwon 642-120, Republic of Korea

^2^ Department of Electrical Functionality Material Engineering, Korea University of Science and Technology (UST), Changwon, 642-120, Republic of Korea

Correspondence and requests for materials should be addressed to J.T.H. (email: jthan@keri.re.kr) or G.-W.L. (email: gwleephd@keri.re.kr)


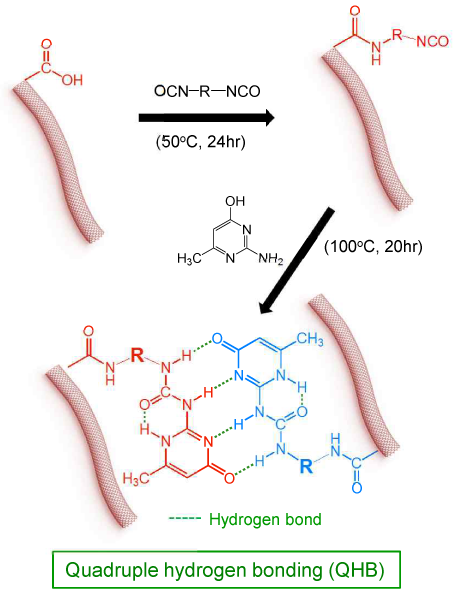


**Figure S1 |** Synthesis scheme of LMWNTs functionalised with quadruple hydrogen bonding motifs by sequential coupling reactions.


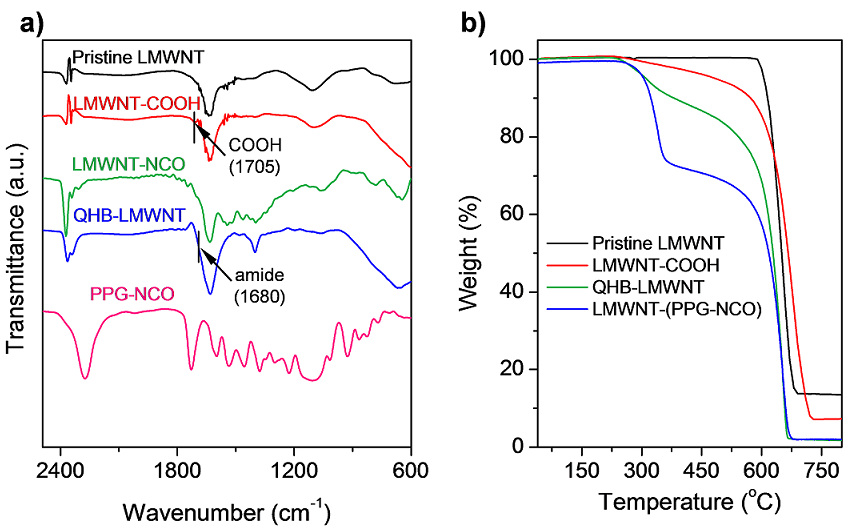


**Figure S2 |** (a) FTIR spectra and (b) thermogravimetric spectra of pristine LMWNTs and LMWNTs functionalized with carboxylic acid (LMWNTs-COOH), isocyanate (LMWNTs-NCO), 2-ureido-4[*1H*]pyrimidinone moieties (QHB-MWNTs), and LMWNTs-COOH reacted with PPG NCO. The band at 1705 cm^–1^ in the FTIR spectra indicates the presence of carboxylic acid groups. This band disappeared and an isocyanate peak (2270 cm^–1^) appeared after the coupling reaction with diisocyanate compounds. Importantly, amide bonds (1680 cm^–1^) and broad hydrogen peaks were observed after the coupling reaction with amino-4-hydroxy-6-methyl-pyrimidine.


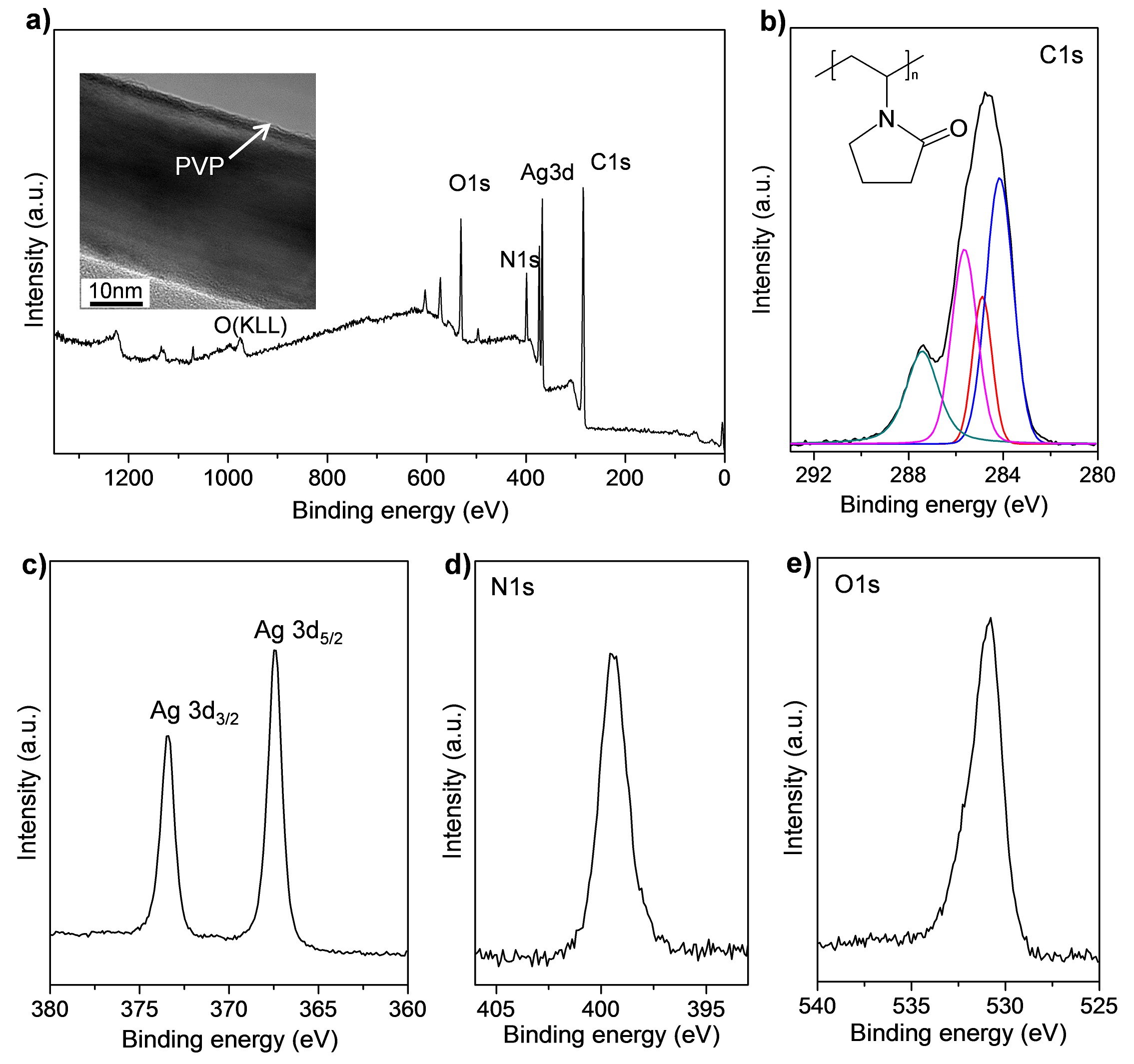


**Figure S3 |** XPS spectra of AgNWs: (a) overall spectrum, (b) C1s, (c) Ag d, (d) N1s, (e) O1s. The inset image in (a) shows TEM image of AgNW showing the width of its diameter and the decorated PVP.


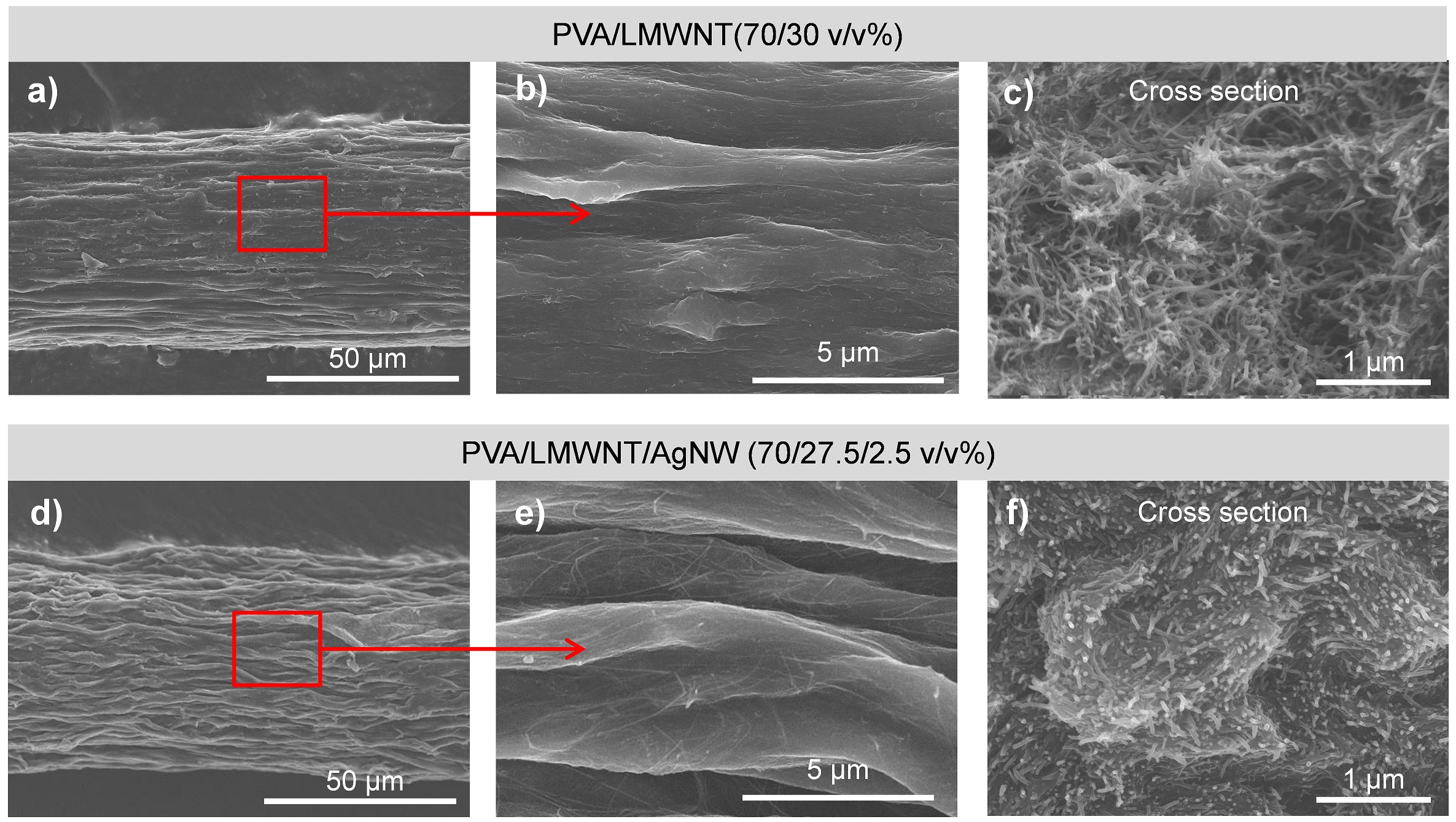


**Figure S4 |** FESEM images of QHB-LMWNT/PVA composite fibre (a - c) and QHB-LMWNT/PVA/AgNW fibre (d-f).


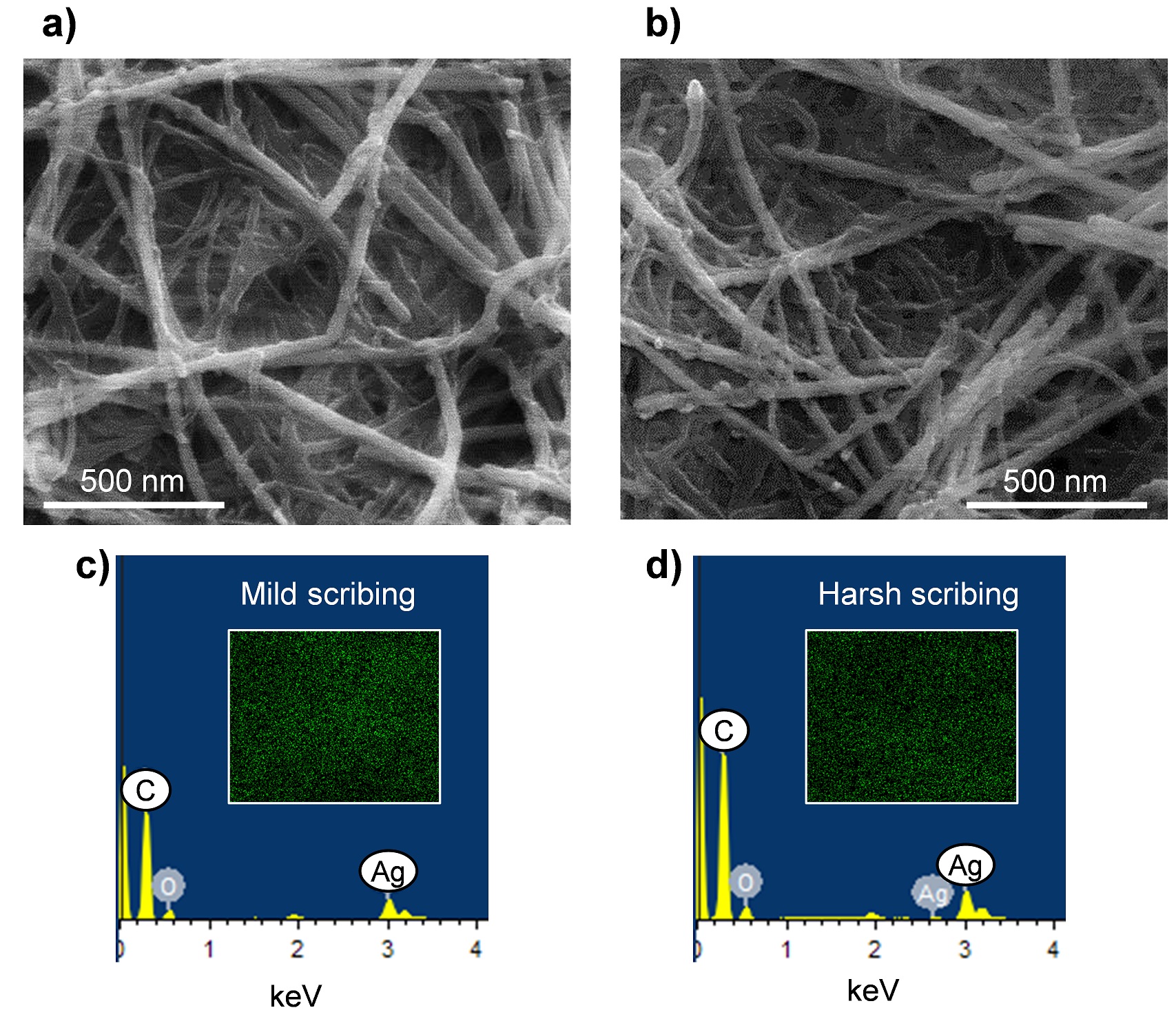


**Figure S5 |** a, b)FESEM images of QHB-LMWNT/PVA/AgNW composite fibres prepared with 5 wt% paste after (a) mild and (b) harsh chemical scribing. c, d) EDX data of corresponding samples in (a) and (b), respectively. Inset images in (a) and (b) show EDX mapping images of Ag atom.


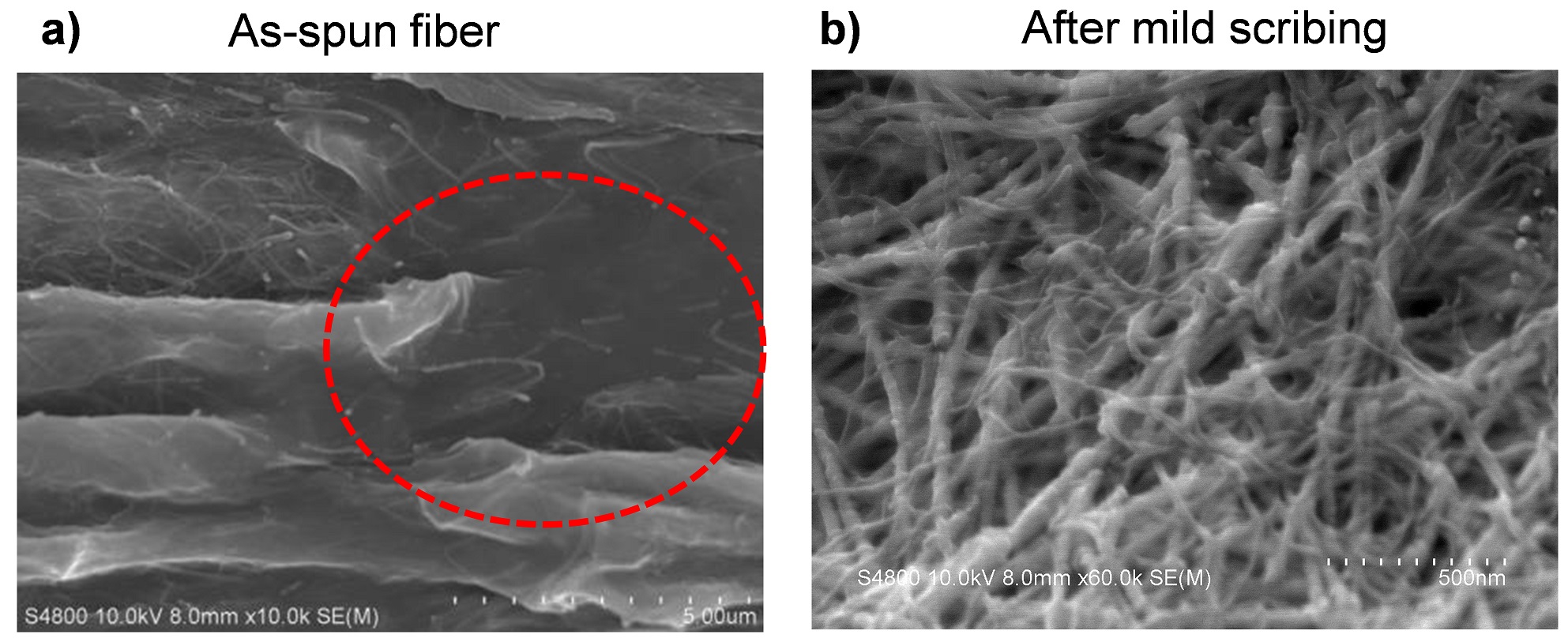


**Figure S6 |** FESEM images of QHB-LMWNT/PVA/AgNW composite fibre prepared by coagulation in methanol for 12hr (a) before and (b) after mild scribing with DMSO/methanol (1/1 v/v) solution. The red circle indicates the PVA rich area on the fibre surface.


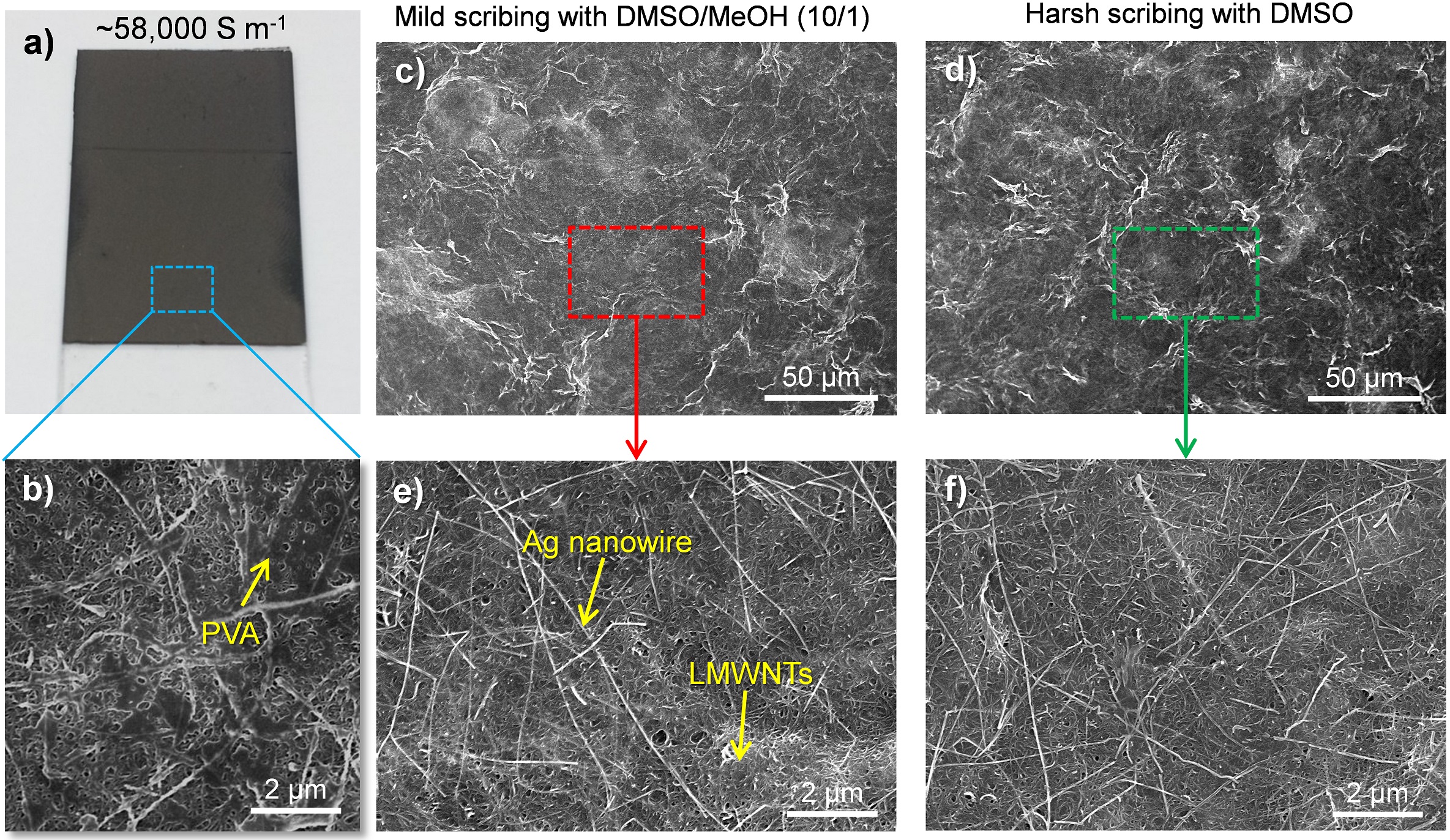


**Figure S7 |** a) Photograph of bar-coated QHB-LMWNT/PVA/AgNW hybrid film containing 2 vol% of AgNWs. b-f) FESEM image of (b) the as-prepared film and that after (c, e) mild scribing and (d, f) harsh scribing. (e) and (f) show the high magnification images of (c), (d), respectively.

**Movie S1:** The touch glove with composite conducting fibres for the touch screen.
